# Urinary Concentrations of Phthalate Metabolites and Bisphenol A and Associations with Follicular-Phase Length, Luteal-Phase Length, Fecundability, and Early Pregnancy Loss

**DOI:** 10.1289/ehp.1408164

**Published:** 2015-07-10

**Authors:** Anne Marie Jukic, Antonia M. Calafat, D. Robert McConnaughey, Matthew P. Longnecker, Jane A. Hoppin, Clarice R. Weinberg, Allen J. Wilcox, Donna D. Baird, Antonia M. Calafat, D. Robert McConnaughey, Matthew P. Longnecker, Jane A. Hoppin, Clarice R. Weinberg, Allen J. Wilcox, Donna D. Baird

**Affiliations:** 1 Epidemiology Branch, National Institute of Environmental Health Sciences (NIEHS), National Institutes of Health (NIH), Department of Health and Human Services (DHHS), Durham, North Carolina, USA; 2 Division of Laboratory Sciences, National Center for Environmental Health, Centers for Disease Control and Prevention, Atlanta, Georgia, USA; 3 Westat, Durham, North Carolina, USA; 4 Department of Biological Sciences, North Carolina State University, Raleigh, North Carolina, USA; 5 Biostatistics Branch, NIEHS, NIH, DHHS, Durham, North Carolina, USA

## Abstract

**Background:**

Certain phthalates and bisphenol A (BPA) show reproductive effects in animal studies and potentially affect human ovulation, conception, and pregnancy loss.

**Objectives:**

We investigated these chemicals in relation to follicular- and luteal-phase lengths, time to pregnancy, and early pregnancy loss (within 6 weeks of the last menstrual period) among women attempting pregnancy.

**Methods:**

Women discontinuing contraception provided daily first-morning urine specimens and recorded days with vaginal bleeding for up to 6 months. Specimens had previously been analyzed for estrogen and progesterone metabolites and human chorionic gonadotropin. A total of 221 participants contributed 706 menstrual cycles. We measured 11 phthalate metabolites and BPA in pooled urine from three specimens spaced throughout each menstrual cycle. We analyzed associations between chemical concentrations and outcomes using linear mixed models for follicular- and luteal-phase lengths, discrete-time fecundability models for time to pregnancy, and logistic regression for early pregnancy loss.

**Results:**

Higher concentrations of monocarboxyoctyl phthalate (MCOP) were associated with shorter luteal phase [2nd tertile vs. 1st tertile: –0.5 days (95% CI: –0.9, –0.1), 3rd vs. 1st: –0.4 days (95% CI: –0.8, 0.01), *p* = 0.04]. BPA was also associated with shorter luteal phase [2nd vs. 1st: –0.8 days (95% CI: –1.2, –0.4), 3rd vs. 1st: –0.4 days (95% CI: –0.8, 0.02), *p* = 0.001].

**Conclusions:**

BPA and MCOP (or its precursors) were associated with shorter luteal phase. Menstrual cycle–specific estimates of urinary BPA and phthalate metabolites were not associated with detrimental alterations in follicular-phase length, time to pregnancy, or early pregnancy loss, and in fact, DEHP [di(2-ethylhexyl) phthalate] metabolites {MEOHP [mono(2-ethyl-5-oxohexyl) phthalate] and ΣDEHP} were associated with reduced early loss. These findings should be confirmed in future human studies.

**Citation:**

Jukic AM, Calafat AM, McConnaughey DR, Longnecker MP, Hoppin JA, Weinberg CR, Wilcox AJ, Baird DD. 2016. Urinary concentrations of phthalate metabolites and bisphenol A and associations with follicular-phase length, luteal-phase length, fecundability, and early pregnancy loss. Environ Health Perspect 124:321–328; http://dx.doi.org/10.1289/ehp.1408164

## Introduction

Human exposure to phthalates is widespread [Centers for Disease Control and Prevention (CDC) 2015]. Phthalates are used to increase the flexibility of certain plastics and can also be found in pharmaceuticals, personal care products, paints, toys, medical devices, and building supplies (CDC 2015). Phthalates may diffuse into their surroundings, and exposure normally occurs through ingestion of contaminated food, dermal application of lotions or perfumes, or inhalation.

Animal studies have suggested that phthalates can decrease fertility and litter size, and interfere with development of the offspring (Gray et al. 2006; Yamasaki et al. 2009). The mechanism for these effects may involve lower production of estradiol or progesterone (Kay et al. 2013). In humans, most studies have focused on pregnancy and offspring outcomes, with fewer studies on intermediate measures of reproductive function.

Recently, mono(2-ethylhexyl) phthalate (MEHP), a metabolite of di(2-ethylhexyl) phthalate (DEHP), was shown to stimulate oxidative stress responses in human placental cells (Tetz et al. 2013). This is further supported by data from the National Health and Nutrition Examination Survey (NHANES) suggesting that several phthalate metabolites are associated with markers of oxidative stress or inflammation (Ferguson et al. 2011, 2012, 2014). In the only published study of naturally conceived pregnancies, urinary MEHP was associated with increased risk of early pregnancy loss in a prospective cohort study of 128 Danish women who had 32 early pregnancy losses (Toft et al. 2012).

Exposure to bisphenol A (BPA), another environmental chemical, is also widespread (CDC 2015). Animal data suggest that BPA reduces female fertility (Berger et al. 2007, 2008, 2010; CDC 2015; Takai et al. 2001; Tsutsui et al. 1998). Evidence suggests that oral BPA exposure disrupts meiosis *in vitro* (Can et al. 2005; Lenie et al. 2008; Machtinger et al. 2011) and *in vivo* (Hunt et al. 2003; Muhlhauser et al. 2009). Among women undergoing *in vitro* fertilization, urinary BPA concentration was associated with reduced peak estradiol, oocyte yield, and oocyte quality (Ehrlich et al. 2012; Mok-Lin et al. 2010). However, data are lacking for women who conceive without intervention.

Phthalates and BPA may reduce fecundability through several mechanisms, including ovulation, conception, and subclinical pregnancy loss. Therefore, we explored possible adverse effects that could be exerted through lengthened follicular phase or shortened luteal phase, increased time to pregnancy, or increased early pregnancy loss. These end points were examined in relation to menstrual cycle–specific urinary concentrations of phthalate metabolites and BPA in a cohort of women who were attempting to become pregnant naturally. Given the previously described literature, we focused on the metabolites of DEHP and BPA as potential risk factors for increased early loss. To our knowledge, this study represents the first study of BPA and early human pregnancy in a cohort of naturally conceiving women.

## Methods

*Study sample.* The North Carolina Early Pregnancy Study (EPS) was a prospective cohort study designed to estimate the incidence of early pregnancy loss (Wilcox et al. 1988b). Briefly, 221 healthy women with no known fertility problems or chronic health conditions were recruited in 1982–1986 from local communities and enrolled at the time they discontinued contraception to become pregnant. An in-person interview at enrollment collected information on women’s demographic, reproductive, medical, and behavioral characteristics, including their age, education, and body weight. Women reported their weekly intake of beer, wine, or hard liquor, caffeinated coffee, tea and iced tea, and caffeinated sodas. Women who were still in the study 3 months after enrollment were asked again about their alcohol and caffeine intake.

Participants collected first-morning urine specimens and completed daily diaries, until a clinical pregnancy was conceived or 6 months had passed with no clinically recognized pregnancy. Nineteen women withdrew from the study before completing the 6-month follow-up. Data collected during their participation are included in these analyses. Urine specimens were collected in 30-mL wide-mouth polypropylene jars with screw tops and without preservatives. They were stored for up to 2 weeks in participants’ home freezers. Samples were transported to a central storage unit in insulated chests with ice packs and stored at –20°C. Specimens were analyzed for reproductive hormones (see below for a detailed description) and then transferred to long-term storage vials (first glass, then polypropylene). Care was taken to keep the samples frozen throughout transport. New aliquots were drawn from these stored samples for phthalate and BPA measurement. Each participant provided written informed consent, and the study protocol was approved by the Institutional Review Board of the National Institute of Environmental Health Sciences. The analysis of blinded specimens by the CDC laboratory was determined not to constitute engagement in human subjects research.

*Outcome measures.* Daily urine specimens were assayed previously for estrone 3-glucuronide (a metabolite of estrogen) and pregnanediol 3-glucuronide (a metabolite of progesterone). These data were used to identify the day of ovulation (or anovulation, if no ovulation was detected) (Baird et al. 1991). Follicular-phase length was defined as the number of days from the first day of menses (based on the bleeding pattern recorded in the daily diary) up to but not including the estimated day of ovulation (*n* = 675). Luteal-phase length was defined as the number of days from the day after ovulation up to, but not including, the first day of the subsequent menses (*n* = 482).

Urine specimens were assayed for human chorionic gonadotropin (hCG) (Wilcox et al. 1988a). Pregnancy was identified by an hCG level exceeding 0.025 ng/mL on 3 consecutive days. If hCG subsequently declined before 6 completed weeks of gestation (42 days), it was considered an early pregnancy loss. There were 48 early pregnancy losses detected and 150 clinical conceptions (conceptions lasting > 6 weeks), of which 15 ended in clinical miscarriage (6–25 gestational weeks).

We measured time to pregnancy as the number of ovulatory menstrual cycles from enrollment to conception (including the conception cycle), with censoring at the time collection was discontinued. The 221 participants in the study contributed a total of 740 menstrual cycles, of which 706 are included in this analysis (see Supplemental Material, Table S1).

*Sample size.* Seven anovulatory cycles were excluded, as were 27 cycles in which there was no unprotected intercourse during the 6-day fertile window (Wilcox et al. 1995). Cycles for which we could not identify a day of ovulation were included if intercourse occurred 12–19 days before the start of the next cycle (*n* = 28). There were 706 cycles in the time-to-pregnancy analysis. The cycles missing day of ovulation (*n* = 28) provided no data on follicular- or luteal-phase lengths, leaving 678 cycles for analysis. Furthermore, because conception interferes with subsequent menstrual bleeding, luteal-phase length could not be defined for the 191 conception cycles, which included 48 early losses. In three women, two ovulations occurred with no reported menses in between. Each ovulation was considered its own cycle; however, the first cycle was missing a luteal-phase length and the second cycle was missing a follicular-phase length, leaving 675 follicular phases and 484 luteal phases. Finally, two women did not have an end date for the last cycle in the study, leaving 482 luteal phases for analysis.

*Phthalate metabolites and BPA measures.* We tested the potential for using stored EPS urine samples to examine associations of BPA and phthalate metabolite concentrations with reproductive outcomes. Published results from those pilot studies (Baird et al. 2010; Nepomnaschy et al. 2009) describe the basis for using EPS urine samples in a full analysis (described here). Briefly, we found that the mean and distribution of BPA were similar to those of the first available NHANES samples collected in 2003–2004, and concentrations of the phthalate metabolites in EPS samples tended to be higher than for the first 1999–2002 NHANES samples (CDC 2015), but the relative ranking of the phthalate metabolites changed little between sampling periods. In addition, the metabolites of DEHP were highly correlated, as they are in samples collected after 2000, such as NHANES (Baird et al. 2010; CDC 2015). Most important, the reproducibility of samples drawn 2 or 4 weeks apart was similar to reproducibility reported for samples collected after 2000. These comparisons suggest long-term stability of both BPA and phthalate metabolites across the past 25 years (Baird et al. 2010; Nepomnaschy et al. 2009).

*Urine specimen selection.* Monday urine specimens were used in most cases because women collected two vials on Mondays, providing twice the volume of sample on those days. However, when those samples were missing (or the cycle was too short), we substituted an available sample from another day of the week. Of the urine specimens used in the pooled samples, 14% were non-Monday specimens (104 of the 706 cycle-specific pooled samples included at least one non-Monday aliquot). Menstrual cycle phase was not associated with urinary phthalate metabolite or BPA concentrations in previous analyses (Baird et al. 2010; Nepomnaschy et al. 2009). Women could have been pregnant when the luteal-phase specimen was collected. However, pregnancies took at least 6 days to implant (most took 8–10 days), so it is unlikely that the pregnancy had implanted in the uterus at the time the sample had been collected. This also suggests that the pregnancy had little time to potentially affect the measured concentrations of BPA or phthalate metabolites. Occasionally women collected their urine specimen later in the day. Of the urine specimens chosen for phthalate metabolite and BPA measurements, only 5% were late voids, and thus we refer to the samples as “first-morning.”

Three urine specimens were chosen from each menstrual cycle, including at least one specimen from the luteal phase. A fixed-volume aliquot was taken from each urine specimen, and the three aliquots from each cycle were pooled to provide a more stable exposure measure summarizing the cycle. After deconjugation, we measured the total urinary concentration of BPA and of phthalate metabolites in the pooled urine sample using online solid phase–extraction, high-performance liquid chromatography–isotope dilution tandem mass spectrometry at CDC (Silva et al. 2007; Ye et al. 2005). To explore the possibility of contamination, we analyzed 10 random individual samples for free and total concentrations of BPA. We did not detect free BPA in 9 of the samples; in 1, the BPA concentrations (free, 2.1 ng/mL; total, 2.9 ng/mL) were suggestive of possible contamination or degradation.

Measurement of phthalate metabolites is preferred to measurement of the parent compounds because contamination of the sample by parent compounds is not expected to significantly increase metabolite levels; also, phthalate metabolites are considered the biologically active molecules (Hauser and Calafat 2005). The phthalate metabolites measured in this study are those typically measured in NHANES and other epidemiologic studies and are listed in Table 1. We calculated the molar sum (in nanomoles per milliliters) of the DEHP metabolites (ΣDEHP): MEHP, mono(2-ethyl-5-oxohexyl) phthalate (MEOHP), mono(2-ethyl-5-hydroxyhexyl) phthalate (MEHHP), and mono(2-ethyl-5-carboxypentyl) phthalate (MECPP). None of the measurements from the pooled specimens were below the limits of detection (LODs). LODs were 0.5 ng/mL for MEHP, 0.4 ng/mL for mono-*n*-butyl phthalate (MnBP), monoethyl phthalate (MEP), and BPA, 0.3 ng/mL for mono-iso-butyl phthalate (MiBP), and 0.2 ng/mL for the remaining metabolites. All measures were standardized by the creatinine concentration measured in the pooled specimen. For comparison, we calculated the median and interquartile range of urinary phthalate metabolites and BPA measured in the 2009–2010 NHANES [National Center for Health Statistics (NCHS) 2011a, 2011b]. The estimates were weighted using the NHANES examination sample weights to produce national estimates and to adjust for the complex sample design of NHANES.

**Table 1:** Phthalate metabolites measured in pooled urine samples from EPS participants.

Phthalate metabolite	Abbreviation	Parent compound	Parent compound abbreviation
Mono-*n*-butyl phthalate	MnBP	Di-*n*-butyl phthalate	DnBP
Monoethyl phthalate	MEP	Diethyl phthalate	DEP
Monobenzyl phthalate	MBzP	benzylbutyl phthalate	BzBP
Mono(2-ethylhexyl) phthalate	MEHP	Di(2-ethylhexyl) phthalate	DEHP
Mono(2-ethyl-5-hydroxyhexyl) phthalate	MEHHP	Di(2-ethylhexyl) phthalate	DEHP
Mono(2-ethyl-5-oxohexyl) phthalate	MEOHP	Di(2-ethylhexyl) phthalate	DEHP
Mono(2-ethyl-5-carboxypentyl) phthalate	MECPP	Di(2-ethylhexyl) phthalate	DEHP
Monocarboxynonyl phthalate	MCNP	Di-isodecyl phthalate	DiDP
Monocarboxyoctyl phthalate	MCOP	Di-isononyl phthalate	DiNP
Mono(3-carboxypropyl) phthalate	MCPP	Di-*n*-octyl phthalate	DOP
		Di-*n*-butyl phthalate	DnBP
Mono-isobutyl phthalate	MiBP	Di-isobutyl phthalate	DiBP

Before carrying out tests of association, we used Akaike’s Information Criterion to decide for each phthalate metabolite and BPA whether to model it in tertiles or as linear, quadratic, or a spline predictor, based on goodness of fit of the unadjusted associations. This vetting procedure led to tertile analysis for all models.

*Analysis.* We analyzed the relation between the natural logarithm of follicular-phase length (to achieve normality) and BPA and phthalate metabolites urinary concentrations (untransformed) measured during a given menstrual cycle using linear mixed models with a random intercept for each woman to account for dependence among cycles within each woman. The same models were used to analyze the association between luteal-phase length (untransformed) and BPA and phthalate metabolite concentrations. We exponentiated the beta estimates from the follicular-phase model (given that it was log-transformed) to obtain the “percent difference” between each of the higher tertiles and the lowest (referent) tertile.

Time to pregnancy is inversely related to fecundability (defined as the probability of conceiving a pregnancy in a given menstrual cycle) (Baird et al. 1986). Time to pregnancy was measured as the number of menstrual cycles until a clinical conception was achieved (it included cycles of early loss, which was studied as a separate outcome). We compared fecundability across cycle-specific concentrations of phthalate metabolites and BPA using fecundability ratios, estimated from a discrete-time hazard model of time to pregnancy (Weinberg and Wilcox 2008). Data were censored at the end of participation for those who had not conceived a clinical pregnancy. This analysis identifies differences in cycle-specific phthalate concentrations for conception cycles compared with nonconception cycles across each cycle of trying (as a “between-women” analysis). We checked the proportional-hazards assumption by testing interactions with time; none had a *p*-value < 0.1. The number of cycles contributed by each woman ranged from 1 to 9 (see Supplemental Material, Table S1); however, only one woman had an eighth and ninth cycle of observation, so these cycles were collapsed down and analyzed with the seventh cycles to avoid inestimable parameters.

In a secondary analysis, we also compared BPA and phthalate metabolite concentrations for nonconception and clinical conception cycles within each woman. This analysis was limited to women who had at least one nonconception cycle and a clinical pregnancy in the study (*n* = 94). These matched sets were analyzed with conditional logistic regression. Given the smaller sample size and the necessary exclusion of the most and least fertile couples, we consider this analysis to be secondary to the between-women analysis.

The associations of conception-cycle phthalate metabolite and BPA concentrations with early pregnancy loss were analyzed using unconditional logistic regression.

For all outcomes, unless otherwise noted, *p*-values represent 2-degrees-of-freedom (df) tests of differences among the tertiles. Linear trend *p*-values were obtained post hoc for exposures showing increasing or decreasing estimates across tertiles. We fitted a linear coefficient to the tertiles and performed a 1-df test on that parameter. In all analyses, *p* ≤ 0.05 was considered statistically significant.

Potential covariates for the analysis of each outcome were chosen considering our previous analyses of EPS data and other literature on these outcomes. The chosen adjustment factors were entered into multivariable models, and only variables that were associated with the outcome (*p* < 0.1) or improved point estimate precision were retained. For follicular-phase length (Jukic et al. 2007), we adjusted for age (continuous), alcohol intake (in three categories based on the 25th and 75th percentiles), and recent use of oral contraceptives (within 90 days of menstrual cycle start). Body mass index (BMI) was not associated with follicular-phase length in the full models, and its exclusion improved precision; thus, BMI was not included in the final models.

The covariates for the analysis of luteal-phase length (Fenster et al. 1999; Windham et al. 2002) were age (continuous) and BMI (in categories: < 20, 20–25, > 25). Caffeine, alcohol, and smoking were not associated with luteal-phase length in the full models and were removed to improve the precision of the estimates.

The covariates for the time-to-pregnancy analysis (Weinberg et al. 1989; Wilcox et al. 1988a) were age, age at menarche (in three categories), smoking at enrollment (yes/no), alcohol intake (in three categories based on the 25th and 75th percentiles), BMI, caffeine consumption (in three categories based on the 25th and 75th percentiles), and education. Alcohol and caffeine use were time-dependent variables, with cycles of attempt of three or more assigned the alcohol and caffeine intake levels reported on the 3-month questionnaire. We found no association of season of the year with fecundability (*p* = 0.83), and season was therefore not included in the multivariable model. *In utero* exposure to maternal smoking was missing for 16% of cycles, and adjustment did not change the presented conclusions, so it was not included in the final models. No covariates were included in the comparison of nonconception and conception cycles within women because we had limited information on time-varying covariates, and the number of women in this analysis was small.

The covariates for the analysis of early pregnancy loss (Weinberg et al. 1994; Wilcox et al. 1990) were age, current smoking, alcohol intake, BMI, caffeine consumption, education, and season of conception. Season of conception was analyzed with trigonometric regression, converting the date of ovulation to radians and calculating the sine and cosine of this angle (Galbraith 2014). Excluding women who reported *in utero* exposure to diethylstilbestrol (*n* = 5) did not substantially alter the estimates, thus these women were included in the final model.

In adult female mice, the adverse effects of environmentally relevant doses of BPA on meiosis were strongest for BPA administration during oocyte growth, which begins 2–3 estrus cycles before ovulation (Hunt and Hassold 2008; Hunt et al. 2003). Oocyte growth in humans is also prolonged, with a mean time of 3.5 cycles from preantral follicle to ovulation (Greenwald and Terranova 1988). For this reason, we performed a sensitivity analysis examining a 1-cycle lag in BPA concentrations—BPA in 1 cycle with luteal-phase length, follicular-phase length, early loss or conception in the subsequent cycle. Additionally, to follow up on our present findings with luteal-phase length, we examined the association of BPA and monocarboxyoctyl phthalate (MCOP) with low mid-luteal progesterone (< 2 μg/mg creatinine). Mid-luteal progesterone was defined as the geometric mean of the urinary progesterone metabolite on days 5 and 6 postovulation (Baird et al. 1999). This measure was associated with reduced fecundability in a previous analysis (Baird et al. 1999). We used generalized estimating equations to estimate the associations of both BPA and MCOP with low mid-luteal progesterone, while accounting for the correlation among cycles within each woman.

## Results

The median age of participants was 29 years (interquartile range, 26–31); most were white (96%), college graduates, parous, and with a BMI of 20–25 kg/m^2^ (see Supplemental Material, Table S2). Few were smokers (*n* = 15). Consumption of alcohol or caffeinated beverages was moderate, with almost 30% of women reporting no alcohol consumption and 50% reporting ≤ 120 mg of caffeine per day.

Compared with NHANES data (2009–2010), women in the EPS (1982–1986) showed higher concentrations of most urinary phthalate metabolites and BPA (Table 2). MEP had the highest concentration in the EPS women, followed by MnBP and the DEHP metabolites. Concentrations of MEHP were higher in the Danish women in 1992–1994 [the prior study that examined early pregnancy loss (Toft et al. 2012)] than in the EPS or NHANES, whereas urinary concentrations of MEHHP and MEOHP were similar to those in the EPS women (Table 2). BPA was not measured in the Danish study.

**Table 2:** Distribution of phthalate metabolites and bisphenol A urinary concentrations in the North Carolina Early Pregnancy Study (EPS) and in comparison populations: U.S. NHANES 2009–2010 and a Danish prospective cohort, 1992–1994 (Toft et al. 2012) [median (IQR)].

Phthalate metabolite or BPA	NHANES^*a*^ (2009–2010) (*n* = 445)	Toft et al.^*b*^ (1992–1994) (*n* = 128)	EPS^*c*^ (1982–1986) (*n* = 706 total menstrual cycles from 221 women)
MnBP	18.2 (7.8, 33.2)	174.1 (106.5, 314.6)^*d*^	80.0 (48.7, 127.0)
MEP	68.0 (24.6, 195.0)	223.6 (113.1, 418.7)	134 (72.4, 296.0)
MBzP	6.7 (3.1, 15.3)	12.4 (7.3, 22.9)	39.5 (24.7, 68.6)
MEHP	1.6 (0.66, 3.3)	15.4 (7.5, 26.6)	6.6 (3.8, 11.2)
MEHHP	11.1 (5.1, 21.9)	36.3 (24.5, 57.4)	50.4 (31.8, 80.8)
MEOHP	7.6 (3.4, 14.8)	33.3 (22.2, 49.5)	30.3 (19.5, 48.9)
MECPP	17.8 (8.9, 32.3)	Not reported	66.0 (42.2, 100.0)
MCNP	2.6 (1.3, 5.3)	Not reported	3.4 (2.1, 6.2)
MCOP	11.2 (4.6, 28.9)	Not reported	3.3 (2.3, 5.2)
MCPP	2.6 (1.3, 5.9)	Not reported	13.5 (8.4, 22.1)
MiBP	9.1 (7.0, 11.2)	Not reported	3.1 (1.9, 5.0)
BPA	1.9 (0.79, 3.9)	Not reported	2.7 (1.8, 4.3)
^***a***^ng/mL, among women 20–44 years of age. ^***b***^For the conception cycle, ng/mL, specific gravity adjusted. ^***c***^ng/mL, not adjusted for creatinine. ^***d***^In the Toft et al. (2012) study MnBP was not differentiated from MiBP; it is thought that most of what is measured is MnBP (G Toft, personal communication).			

**Figure 1:**
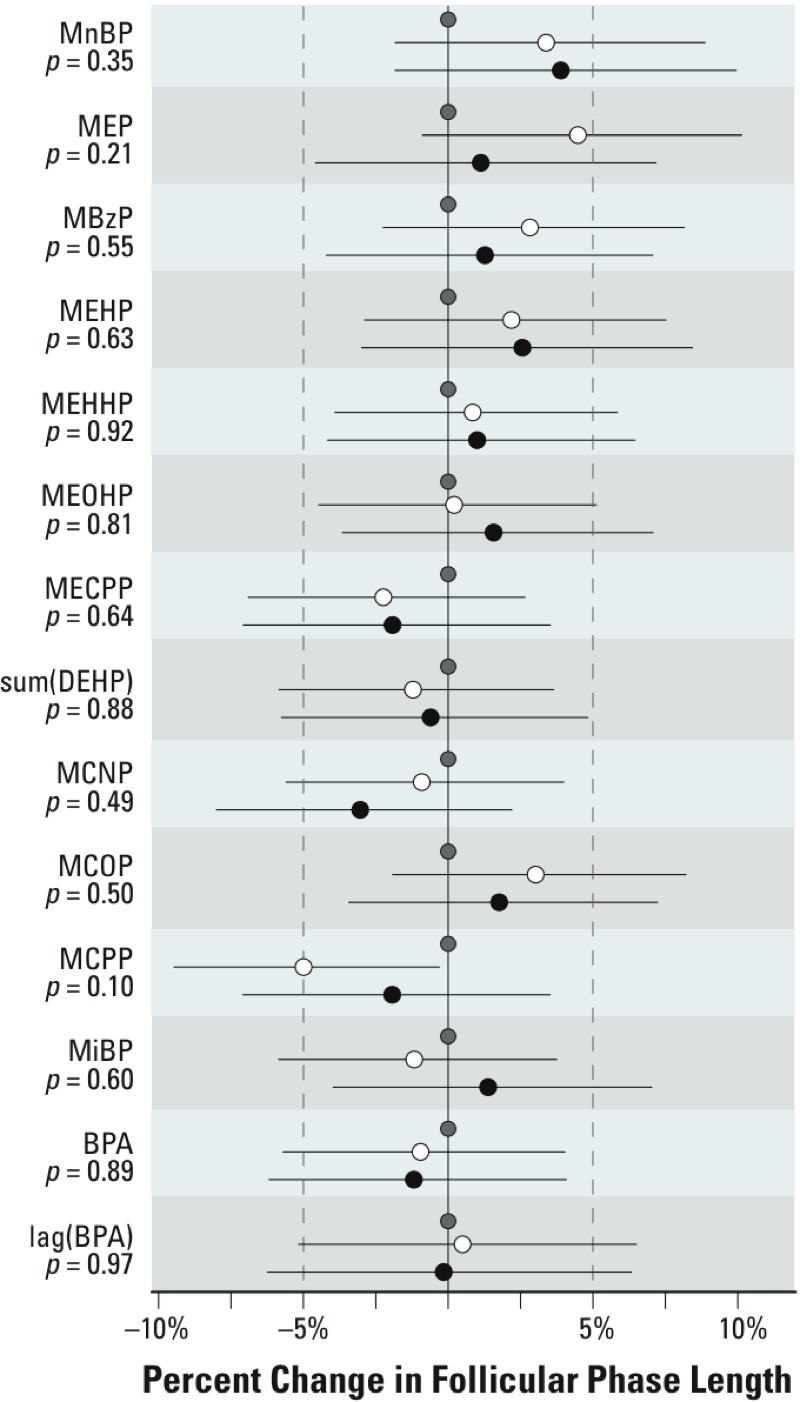
Results of the linear mixed-model regression estimating the associations between urinary concentrations of phthalate metabolites and BPA and the natural log of follicular-phase length among all study cycles (*n* = 706) (adjusted for age, recent oral contraceptive use, and alcohol use). Beta coefficients from the regression model have been exponentiated, resulting in estimates of the relative length of the follicular phase for concentrations in the middle (white circles) and highest (black circles) tertiles compared with the lowest tertile (gray circles). Line segments represent the 95% CI. *p*-Values represent a 2-df test of difference among the tertiles. sum(DEHP) represents the molar sum of four of the metabolites of DEHP: MEHP, MEHHP, MEOHP, and MECPP.

*Follicular-phase length.* Neither BPA nor any of the phthalate metabolites was associated with follicular-phase length (*p* > 0.05, Figure 1). The strongest association was for mono(3-carboxypropyl) phthalate (MCPP, a nonspecific metabolite of several phthalates). The percent change in follicular-phase length [95% confidence interval (CI)], for the second tertile versus the first was –5% (95% CI: –9, 0), and for the third tertile versus the first was –2% (95% CI: –7, 4) (differences among tertiles, *p* = 0.1; Figure 1).

**Figure 2:**
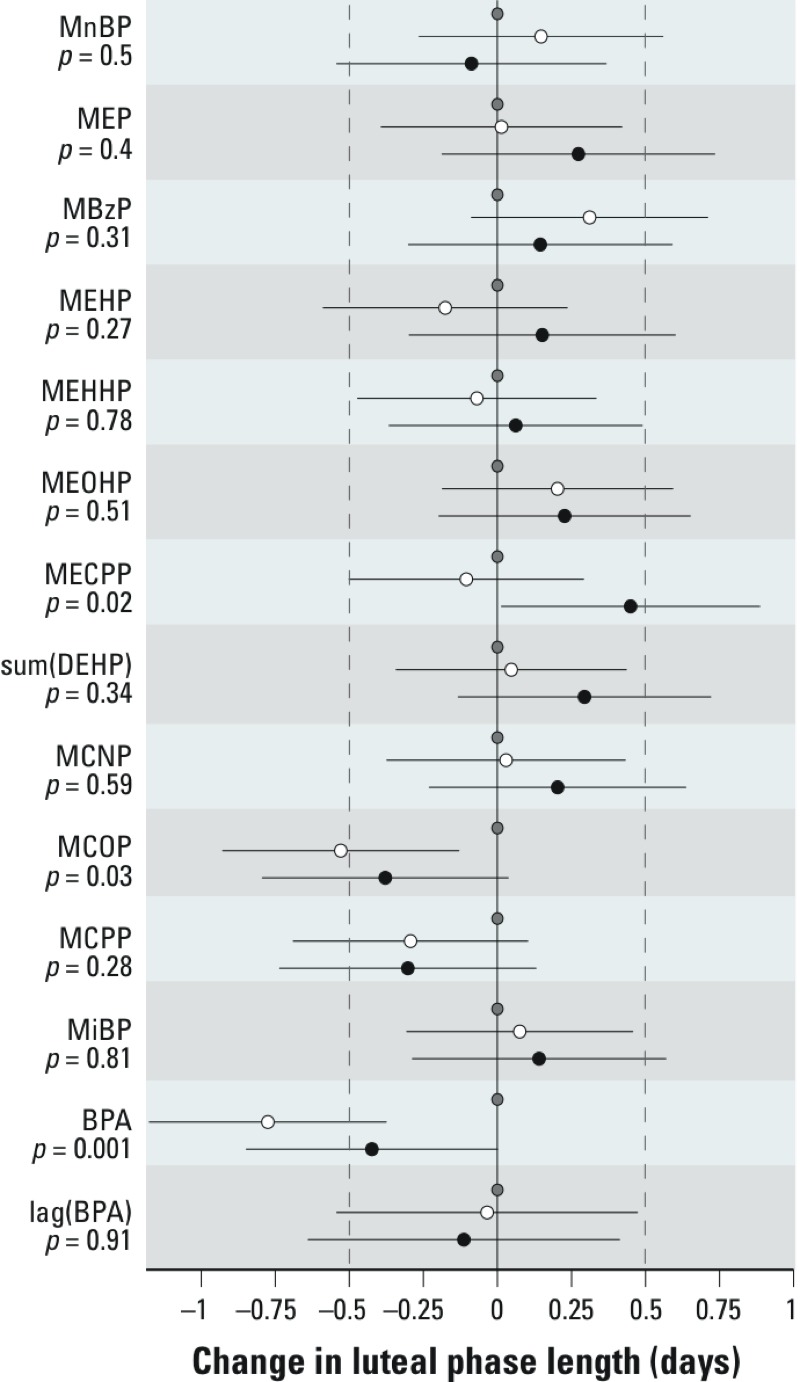
Results of the linear mixed-model regression estimating the associations between urinary concentrations of phthalate metabolites and BPA and luteal-phase length among all study cycles (*n* = 482) (adjusted for age and BMI). Beta coefficients estimate the difference in luteal-phase length for concentrations in the middle (white circles) and highest (black circles) tertiles compared with the lowest tertile (gray circles). Line segments represent the 95% CI. *p*-Values represent a 2-df test of difference among the tertiles. sum(DEHP) represents the molar sum of four of the metabolites of DEHP: MEHP, MEHHP, MEOHP, and MECPP.

*Luteal-phase length.* Two phthalate metabolites and BPA were significantly associated with luteal-phase length (Figure 2). For MECPP, the third tertile showed significantly longer luteal phases than the second, but neither differed significantly from the first tertile. Higher urinary concentrations of MCOP, a metabolite of di-isononyl phthalate (DiNP), were associated with shorter luteal phases by about half a day [second tertile vs. first tertile: –0.5 days (95% CI: –0.9, –0.1), third vs. first: –0.4 days (95% CI: –0.8, 0.01), *p* = 0.03] (Figure 2). Higher BPA concentration was also associated with shorter luteal phases [second vs. first: –0.8 days (95% CI: –1.2, –0.4); third vs. first: –0.4 days (95% CI: –0.8, 0.02), *p* = 0.001]. BPA in the previous cycle was not associated with luteal-phase length (*p* = 0.91).

To further explore associations with shorter luteal phases, we examined the association of BPA and MCOP with low progesterone (< 2 μg/mg creatinine) in the mid-luteal phase. Among 423 cycles (from 154 women) with measured mid-luteal progesterone, 76 (18%) had low concentrations. Elevated BPA was associated with reduced odds of low progesterone [second tertile vs. first, odds ratio (OR) = 0.64 (95% CI: 0.41, 1.0), third tertile vs. first, OR = 0.67 (95% CI: 0.41, 1.1)]; however, it was not statistically significant (*p* = 0.24). There was no association for MCOP [second tertile vs. first, OR = 0.97 (95% CI: 0.58, 1.6), third tertile vs. first, OR = 1.1 (95% CI: 0.6, 2.1), *p* = 0.81].

**Figure 3:**
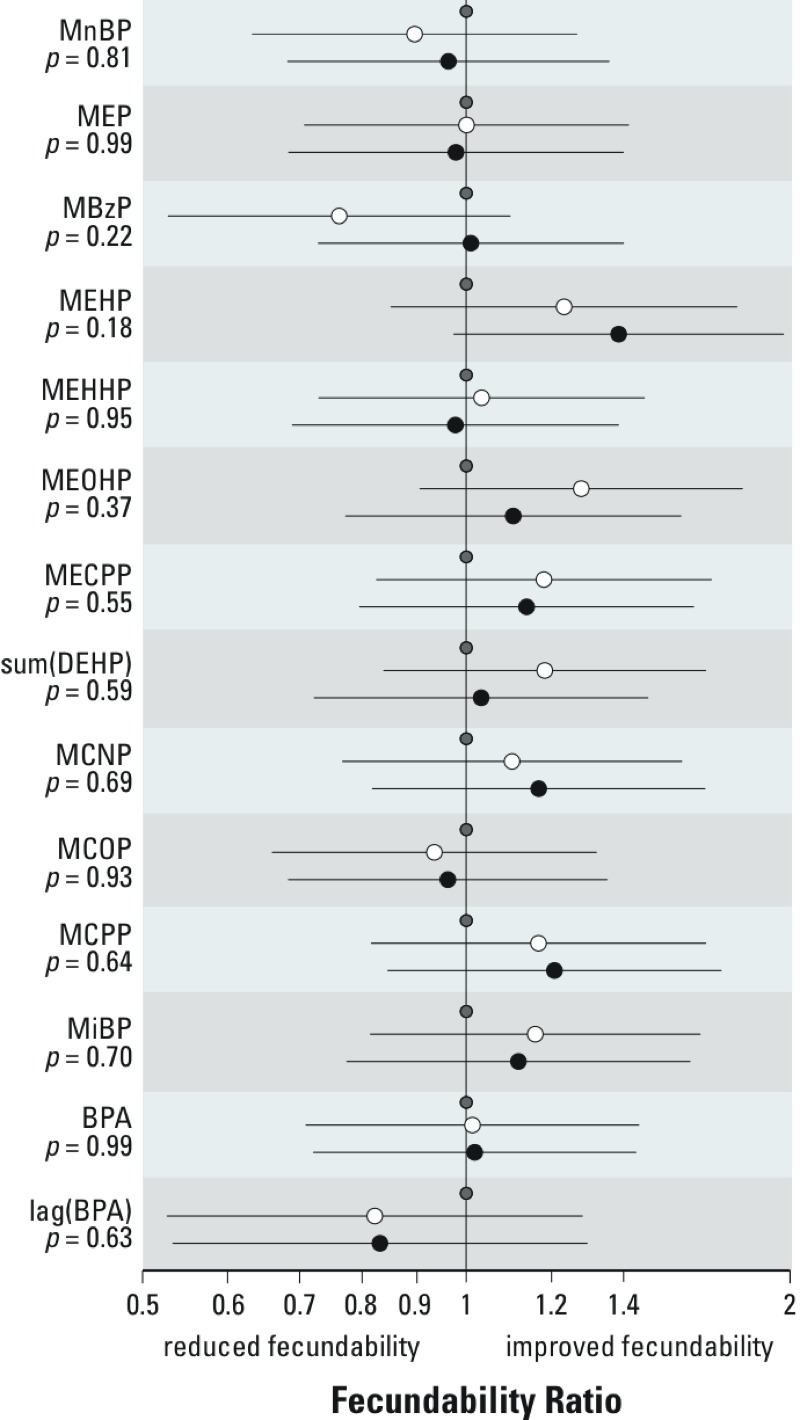
Results of the discrete-time, time-to-event model estimating the associations between tertiles of urinary concentrations of phthalate metabolites, BPA, and fecundability (adjusted for age, age at menarche, current smoking, alcohol intake, BMI, caffeine consumption, and education). The graph shows fecundability ratios for concentrations in the middle (white circles) and highest (black circles) tertiles compared with the lowest tertile of each phthalate (or BPA) (gray circles). Fecundability ratios < 1 correspond to estimated reductions in fecundability. Line segments represent the 95% CI. *p*-Values represent a 2-df test of difference among the tertiles. sum(DEHP) represents the molar sum of four of the metabolites of DEHP: MEHP, MEHHP, MEOHP, and MECPP. The linear trend *p*-value for MEP is 0.91 and for MEHP is 0.07.

*Fecundability (estimated by time to clinical pregnancy).* Estimated associations between tertiles of urinary phthalate metabolite concentrations and fecundability are depicted in Figure 3. A fecundability ratio < 1 indicates reduced fecundability for an “exposed” group. Neither BPA nor any of the phthalate metabolites was associated with reduced fecundability (all *p*-values > 0.05). The strongest association was for MEHP (*p* = 0.18), which showed increased fecundability in the two highest tertiles of MEHP concentrations (but not with the other three DEHP metabolites).

A secondary analysis comparing nonconception cycles with clinical conception cycles in the same woman was conducted. Within-woman comparisons might be more powerful than between-woman comparisons if they eliminate extraneous between-woman variability in exposure metabolism factors. High concentrations of MnBP were statistically significantly associated with nonconception (linear trend *p*-value across tertiles = 0.01). The OR for conception comparing the second tertile of MnBP to the first tertile was 0.6 (95% CI: 0.3, 1.3), and for the third tertile compared with the first was 0.3 (95% CI: 0.1, 0.8). Higher ΣDEHP was also statistically significantly associated with nonconception: second tertile versus first, OR = 0.9 (95% CI: 0.5, 1.7) and third tertile versus first, OR = 0.4 (95% CI: 0.2, 1.0), linear trend *p*-value across tertiles = 0.04. MECPP and MEHHP showed borderline associations with nonconception. For MECPP the OR comparing the second tertile with the first was 1.0 (95% CI: 0.5, 2.0), and for the third tertile was 0.5 (95% CI: 0.2, 1.1); the linear trend *p*-value across tertiles was 0.08. The OR for the second tertile of MEHHP compared with the first tertile was 0.9 (95% CI: 0.5, 1.6) and for the third tertile versus the first was 0.5 (95% CI: 0.2, 1.2); the linear trend *p*-value across tertiles was 0.1.

**Figure 4:**
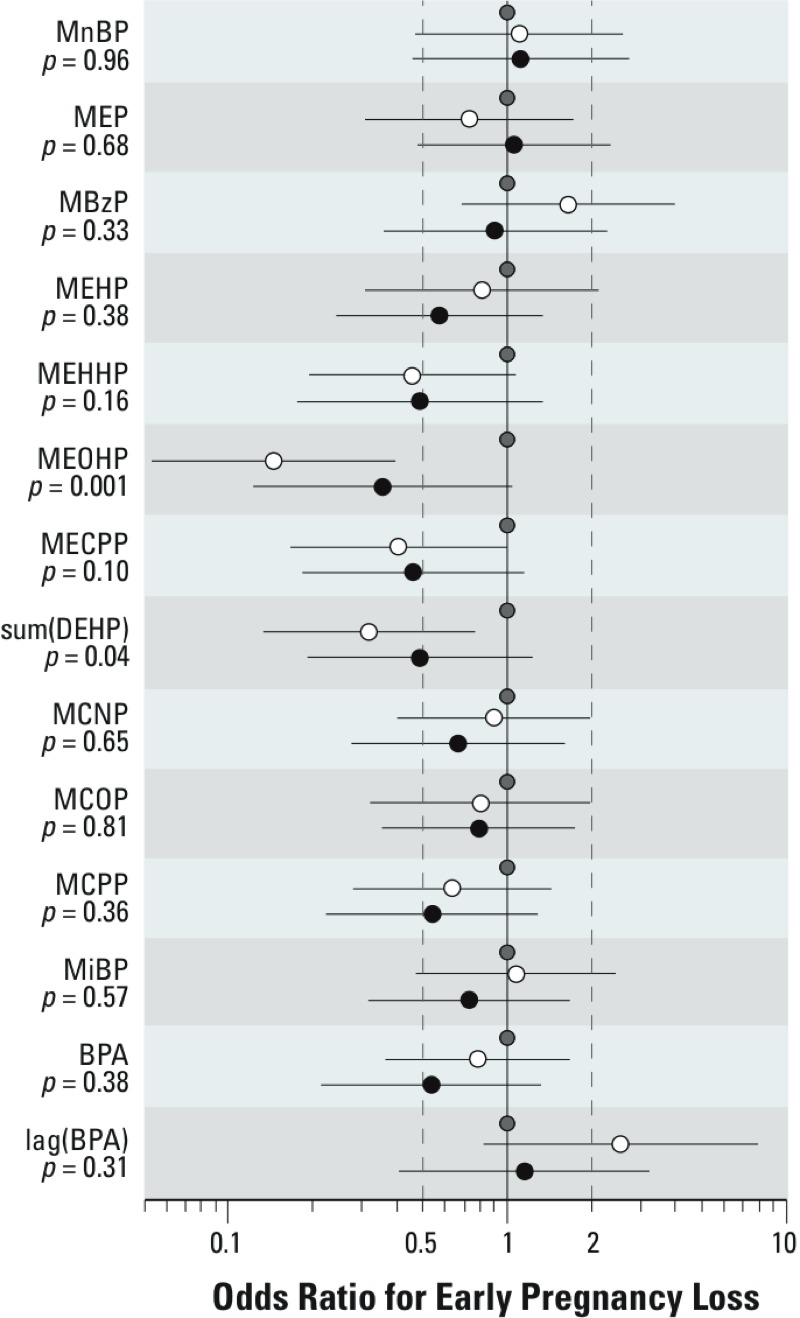
Results of the logistic regression estimating the associations between urinary concentrations of phthalate metabolites and BPA and early pregnancy loss (adjusted for age, current smoking, alcohol intake, BMI, caffeine consumption, education, and season). The two estimates shown for each metabolite are estimates for the middle (white circles) and highest (black circles) tertiles of phthalate or BPA urinary concentration, relative to the lowest tertile (gray circles). Line segments represent the 95% CI. *p*-Values represent a 2-df test of difference among the tertiles. sumDEHP represents the molar sum of four of the metabolites of DEHP: MEHP, MEHHP, MEOHP, and MECPP. Linear trend *p*-values: MEHHP = 0.14, MEOHP = 0.05, MCPP = 0.17, MECPP = 0.10, ΣDEHP = 0.13, and for BPA is 0.17.

*Early pregnancy loss.* None of the phthalate metabolites was associated with increased risk of early pregnancy loss (Figure 4) conditional on conception. Contrary to our *a priori* hypotheses, lower risk of early loss was seen with several urinary DEHP metabolites. Higher ΣDEHP was significantly associated with reduced odds of early loss (*p* = 0.04), whereas the DEHP metabolite MEOHP showed the strongest such association (*p* = 0.001); however, all metabolites of DEHP showed the same direction of higher concentrations being associated with reduced odds of early pregnancy loss. None of these compounds was related to clinical pregnancy loss, although we had limited power to test these associations (15 clinical losses; data not shown).

## Discussion

We found that increased urinary concentrations of BPA were associated with shorter luteal phase. Short luteal phases are less likely to support a conception, should one occur. However, we did not observe lower mid-luteal progesterone, increased time to pregnancy, or early loss with urinary BPA concentrations—end points that would also be expected to be affected by a short luteal phase. In adult female mice, the adverse effects of BPA on meiosis were strongest for BPA administration during oocyte growth, which begins 2–3 estrus cycles before ovulation (Hunt and Hassold 2008). We examined BPA in the cycle before as the exposure of interest to capture that potentially vulnerable time, but none of the outcomes was associated with this lagged BPA.

The time-to-pregnancy analysis showed no associations of BPA or phthalate metabolites with fecundability. However, when nonconception cycles were compared with the clinical conception cycle from the same woman (a secondary, within-woman analysis), MnBP and ΣDEHP were higher in the nonconception cycles. Di-*n*-butyl phthalate (DnBP), the parent compound of MnBP, is a solvent used in personal care products, pharmaceutical coatings, and insecticides (CDC 2015), and exposure to DnBP in animals was associated with reduced litter size, although there were no changes in estrus cyclicity (National Toxicology Program/National Institute of Environmental Health Sciences 1997).

The primary time-to-pregnancy analysis used the data from nearly all our study menstrual cycles. It compared women who conceived a clinical pregnancy with those still trying at each given menstrual cycle (a between-women analysis). It is possible that between-women differences in timing of daily exposures and other habits might result in systematic differences in first-morning urine concentrations among women, even assuming similar levels of exposure, whereas within-woman differences between cycles might be more likely to reflect real cycle-specific differences in exposure.

On the other hand, the within-woman analysis comparing nonconception to conception cycles is based on a small, select sample. Women in this analysis had at least one nonconception cycle and then had conceived a clinical pregnancy during the study. Thus, the most fecund (women who conceived in the first cycle), and the least fecund [women who never conceived or conceived after leaving the study (usually after cycle 6)] were excluded from the within-woman analysis. Given the short half-life of phthalates and BPA, and the likely episodic nature of the exposures, further studies that can examine both within-woman and between-women differences will be needed. We know of only one other between-women analysis of phthalates and BPA and fecundability (Buck Louis et al. 2014). This study found no association of women’s BPA and phthalate metabolite urinary concentrations with fecundability, but did find reduced fecundability with several phthalate metabolites measured in their male partners.

A previous study of Danish women reported increased risk of early pregnancy loss with higher urinary concentrations of the DEHP metabolite MEHP (OR = 41 (95% CI: 4.5, 370) (Toft et al. 2012). This association was not present in our study (highest tertile vs. lowest: OR = 0.5 (95% CI: 0.2, 1.3) though we had approximately 100% power to detect the reported OR of 41. On the contrary, ΣDEHP was associated with reduced odds of early pregnancy loss. It is possible that exposure to DEHP may cause losses to occur earlier, before the detection of hCG in maternal urine, resulting in a net decrease in the frequency of observed early loss.

The findings in the Toft study should be considered critically (Toft et al. 2012). During this study, women were enrolled when they discontinued birth control and were followed prospectively for 6 months or until they conceived a clinical pregnancy. The women collected daily urine specimens for the first 10 days of the menstrual cycle. Of the 430 women enrolled, 77 did not collect any urine samples because they conceived in the first menstrual cycle. The final sample size of Toft et al. (2012) is slightly smaller than ours (128 women who had 32 early pregnancy losses), and it lacked samples from the highly fertile couples who conceived in the first cycle. As Toft et al. (2012) noted, the proportion of pregnancies ending in early loss in the lowest tertile of urinary MEHP concentration was 3%, whereas the overall rate of early loss was 25%, similar to that of the EPS (24%). Thus, the “high” loss in the highest MEHP tertile was attributable to an extremely low loss rate in the referent category. However, the reported urinary concentrations of MEHP were higher in the Danish women in 1992–1994 than in the North Carolina women in 1982–1986, though the Danish women’s urinary concentrations of MEHHP and MEOHP were similar to those in our study (Table 2). The similar urinary concentrations of these oxidative metabolites of DEHP (another oxidative metabolite, MECPP, was not measured in the Danish cohort) in the Danish women and in the EPS women suggests comparable exposures to the parent compound DEHP (Enke et al. 2013). Reasons for the three-times-higher concentrations of MEHP among the Danish women are unknown.

The strengths of our study include hormonally defined ovulation and early pregnancy loss, detailed prospective time-to-pregnancy data, and a study cohort comprising naturally cycling women with no known fertility problems. The use of pooled daily urine specimens from three time points in each menstrual cycle serves to decrease measurement variability and improve precision for assessing exposure effects, given the short half-life of phthalates and BPA. To our knowledge, this is the first study to estimate exposure for each menstrual cycle of interest rather than use an estimate based on a single-day measure.

Our study also has weaknesses. We used mostly Monday-morning urine specimens because women collected two samples every Monday, so that sample was usually available and relatively untouched. However, to the extent that phthalate exposures from cosmetics may vary across the days of the week, our Monday samples may be unrepresentative. The storage containers for EPS urines were unlikely to be sources of contamination by exogenous BPA or phthalates, but we cannot completely rule out contamination or degradation of the urine specimen. If contamination occurred either during urine transfer or processing, its impact would be less problematic for phthalates than for BPA because we measured phthalate metabolites and not the parent compounds. Other studies that report contamination show much higher levels of urinary BPA [7 ng/mL compared with our median of 2.7 ng/mL (Guidry et al. 2015)], which further suggests that widespread BPA contamination of our samples is unlikely. We did not thoroughly explore the associations between phthalate metabolites and BPA with hormone levels because hormone measures were done in restricted time windows and only extensively for a subset of the cycles. Although unique, our study is relatively small, incorporating data from 221 women. These women were healthy volunteers, which may limit generalizability of our findings. Finally, given the number of statistical analyses performed, at least some of the positive associations reported may have arisen by chance.

A final strength of the study is that samples were collected at a time when the U.S. population’s exposures to BPA and phthalates were generally higher than they are now. Urinary concentrations of DEHP metabolites from a recent nationally representative sample of U.S. reproductive-age women (2009–2010 NHANES) are lower than in the EPS, reflecting the reported decline in exposure of certain phthalates in the United States over time (Zota et al. 2014). Other more recent studies also show lower urinary levels than found in our study. Data from the Longitudinal Investigation of Fertility and the Environment cohort found lower levels of all phthalate metabolites (except MiBP) compared with our cohort (Buck Louis et al. 2014). Other U.S. cohorts from the Midwest and California (1999–2005) (Kobrosly et al. 2014), southwest Ohio (2003–2006) (Yolton et al. 2011), and Massachusetts (2004–2009) (Braun et al. 2012) report lower levels of BPA and phthalate metabolites compared with the EPS women, except for MiBP, which was higher. The Norwegian Mother and Child Cohort Study (MoBa) reported lower levels of phthalates compared with the EPS women except for MiBP and MEHP (Ye et al. 2009). The Generation R study reported lower levels of BPA and all phthalates except for MiBP (Ye et al. 2008).

## Conclusions

To our knowledge, this is the first study to examine the association between urinary concentrations of BPA and phthalate metabolites with follicular- and luteal-phase length. Previous data on fecundability and early loss are limited to a single study each (Buck Louis et al. 2014; Toft et al. 2012). The within-woman analysis of nonconception compared with conception cycles raises questions about possible adverse effects of exposure to DEHP and DnBP, the parent compound of MnBP. However, these findings need confirmation because none of the urinary concentrations of phthalate metabolites or BPA were associated with decreased fecundability in the cohort as a whole. In our analyses we found little evidence to suggest that the urinary concentrations of phthalate metabolites we measured or BPA are associated with follicular-phase length or increased risk of early pregnancy loss, but there was an association between ΣDEHP and reduced risk of early loss. Urinary concentrations of BPA and MCOP may be associated with a shorter luteal phase, and this should be confirmed in future studies.

## Supplemental Material

(155 KB) PDF
