# Supplementary material for: Urinary Concentrations of Phthalate Metabolites and Bisphenol A and Associations with Follicular-Phase Length, Luteal-Phase Length, Fecundability, and Early Pregnancy Loss
Source: Environ Health Perspect. 2015 Jul 10;124(3):321–8. doi: 10.1289/ehp.1408164 (PMC4786975; doi:10.1289/ehp.1408164)
Supplement: (155 KB) PDF [file ehp.1408164.s001.acco.pdf]

Environ Health Perspect
DOI: 10.1289/ehp.1408164

Note to Readers: *EHP* strives to ensure that all journal content is accessible to all readers. However, some figures and Supplemental Material published in *EHP* articles may not conform to 508 standards due to the complexity of the information being presented. If you need assistance accessing journal content, please contact ehp508@niehs.nih.gov. Our staff will work with you to assess and meet your accessibility needs within 3 working days.

Supplemental Material

Urinary Concentrations of Phthalate Metabolites and Bisphenol A and Associations with Follicular-Phase Length, Luteal-Phase Length, Fecundability, and Early Pregnancy Loss

Anne Marie Jukic, Antonia M. Calafat, D. Robert McConnaughey, Matthew P. Longnecker, Jane A. Hoppin, Clarice R. Weinberg, Allen J. Wilcox, and Donna D. Baird

Table of Contents

Table S1. Number of cycles contributed by each woman in the time to pregnancy analysis (706 cycles total).

Table S2. Urinary concentrations of phthalate metabolites by demographic and behavioral characteristics for the 221 women enrolled in the Early Pregnancy Study, 1982-1985.

Table S1. Number of cycles contributed by each woman in the time-to-pregnancy analysis (706 cycles total).

Cycles	N (%)
1	62 (28)
2	41 (19)
3	39 (18)
4	19 (9)
5	15 (7)
6	23 (10)
7	21 (10)
9	1 (0.5)

Table S2. Urinary concentrations of phthalate metabolites by demographic and behavioral characteristics for the 221 women enrolled in the Early Pregnancy Study, 1982-1985.

	N (%)	MEP	MnBP	MBzP	MEHP	MEHHP	MEOHP	MCNP	MCOP	MCPP	MECPP	MiBP	BPA
		Mean (se) (ng/mg creatinine)											
Age (years)													
21 - 27	88 (40)	158 (19)	75 (5)	49 (6)	7 (0.9)	49 (9)	30 (5)	5 (0.8)	4 (0.5)	13 (1)	63 (8)	4 (1)	3 (0.3)
28 - 30	62 (28)	257 (47)	106 (33)	37 (3)	10 (2)	70 (10)	41 (6)	5 (0.8)	3 (0.1)	11 (0.8)	89 (13)	5 (1)	2 (0.2)
31 – 42	71 (32)	251 (93)	113 (32)	43 (7)	7 (0.9)	52 (6)	31 (3)	4 (0.6)	3 (0.3)	14 (1)	66 (6)	4 (0.6)	2 (0.2)
Education													
Some college, or less	63 (29)	233 (43)	113 (35)	42 (4)	8 (0.8)	49 (6)	29 (3)	5 (0.7)	3 (0.2)	13 (1)	62 (5)	6 (2)	2 (0.2)
College graduate	84 (38)	277 (81)	74 (7)	42 (6)	8 (1)	58 (10)	36 (6)	4 (0.7)	3 (0.2)	12 (1)	72 (10)	3 (0.5)	3 (0.2)
At least some graduate school	74 (33)	131 (14)	105 (27)	47 (7)	9 (1)	60 (8)	36 (5)	5 (0.7)	3 (0.6)	13 (1)	78 (10)	4 (0.7)	3 (0.2)
Parity													
0	107 (48)	175 (21)	103 (20)	50 (7)	7 (0.7)	50 (7)	29 (4)	4 (0.6)	3 (0.4)	13 (1)	61 (6)	5 (1)	3 (0.2)
1	86 (39)	284 (81)	95 (26)	39 (3)	8 (1)	58 (7)	34 (4)	6 (0.8)	3 (0.2)	12 (0.9)	74 (8)	3 (0.4)	3 (0.2)
>=2	28 (13)	159 (24)	70 (6)	36 (4)	10 (3)	73 (17)	44 (10)	3 (0.8)	3 (0.2)	12 (1)	100 (23)	5 (2)	2 (0.2)
Smoking status													
Current	15 (7)	152 (35)	79 (12)	37 (9)	8 (2)	47 (9)	29 (6)	4 (1)	2 (0.2)	10 (1)	61 (11)	8 (5)	2 (0.4)
Former	52 (24)	197 (46)	112 (42)	35 (3)	9 (1)	74 (16)	43 (9)	6 (1)	3 (0.2)	14 (2)	87 (14)	3 (0.4)	3 (0.3)
Never	154 (70)	228 (45)	92 (14)	47 (5)	8 (0.8)	51 (5)	30 (3)	4 (0.5)	3 (0.3)	12 (0.7)	67 (6)	4 (0.7)	3 (0.2)
Body mass index (kg/m^2)													
≤ 20	81 (37)	222 (38)	101 (27)	48 (8)	8 (0.9)	55 (7)	32 (4)	4 (0.7)	3 (0.2)	13 (1)	69 (7)	4 (0.9)	3 (0.2)
>20 - 25	115 (52)	224 (58)	96 (18)	44 (4)	8 (1.0)	57 (8)	35 (4)	5 (0.6)	3 (0.4)	13 (0.9)	73 (8)	5 (0.8)	3 (0.2)
>25	25 (11)	158 (35)	80 (15)	32 (3)	7 (2)	54 (15)	30 (7)	5 (0.9)	4 (0.4)	11 (1)	70 (17)	4 (2)	3 (0.2)
Caffeine intake (mg/month)													
≤ 1480	62 (28)	262 (105)	79 (8)	40 (5)	6 (0.9)	40 (4)	24 (2)	4 (0.8)	3 (0.2)	11 (0.7)	53 (4)	4 (1)	2 (0.2)
>1480 - <6900	111 (50)	185 (28)	112 (27)	48 (6)	9 (1)	66 (9)	39 (5)	5 (0.7)	3 (0.2)	13 (1)	81 (9)	4 (0.6)	3 (0.2)
≥ 6900	48 (22)	225 (37)	80 (7)	40 (4)	7 (1)	54 (7)	33 (4)	4 (0.7)	4 (0.9)	13 (1)	72 (8)	4 (2)	3 (0.3)
Alcohol intake (servings/week)													
0	57 (26)	302 (114)	113 (36)	38 (4)	8 (1)	46 (6)	28 (3)	5 (1)	3 (0.2)	12 (1)	62 (8)	5 (1)	2 (0.2)
1 - 18	113 (51)	155 (17)	93 (20)	44 (5)	8 (1)	59 (8)	35 (5)	5 (0.6)	3 (0.4)	12 (0.8)	77 (9)	4 (0.8)	3 (0.2)
>18	51 (23)	255 (57)	82 (5)	50 (9)	8 (1)	60 (9)	35 (5)	5 (0.8)	3 (0.3)	14 (2)	69 (7)	4 (0.8)	3 (0.3)
Used oral contraceptives within 90 days of menstrual cycle start													
Yes	33 (15)	173 (29)	81 (10)	40 (5)	7 (2)	59 (23)	35 (12)	6 (1)	3 (0.3)	11 (1)	70 (19)	5 (2)	3 (0.4)
No	184 (85)	226 (40)	98 (16)	45 (4)	8 (0.7)	56 (5)	33 (3)	4 (0.4)	3 (0.3)	13 (0.7)	71 (5)	4 (0.5)	3 (0.1)
